# Lignin-based controlled-release urea improves choy sum growth by regulating soil nitrogen nutrients and bacterial diversity

**DOI:** 10.3389/fpls.2024.1488332

**Published:** 2024-12-04

**Authors:** Xiaojuan Chen, Bosi Lu, Bowen Lv, Shaolong Sun

**Affiliations:** ^1^ College of Agriculture, Guangxi University, Nanning, Guangxi, China; ^2^ Guangxi Key Laboratory for Agro-Environment and Agro-Products Safety, Nanning, China; ^3^ College of Natural Resources and Environment, South China Agricultural University, GuangDong Engineering Technology Research Center of Green Inputs for Low-carbon Agriculture, Guangzhou, Guangdong, China

**Keywords:** lignin-based controlled-release urea, choy sum, growth, soil nitrogen nutrient, bacterial diversity

## Abstract

Lignin, as one of the few renewable resources among aromatic compounds, exhibits significant potential for applications in the agricultural sector. Nonetheless, there has been relatively limited research on the effects of lignin-based controlled-release urea (LCRU) on soil nitrogen nutrition and bacterial diversity. In this paper, the impact of LCRU on the growth of choy sum was investigated through a two-season field experiment. The findings suggest that the plant height, stem diameter, SPAD value, and above-ground dry weight under LCRU application surpassed those with conventional urea (CU), increasing by 40.27%, 26.97%, 52.02%, and 38.62%, respectively. Furthermore, the condition that the urea content was reduced by 15% (LCRU15) caused improvements of 24.76%, 26.97%, 43.23%, and 30.86% in the respective variables. Additionally, compared with the CU, the contents of vitamin C, soluble sugar, and soluble protein in choy sum were increased by the LCRU and LCRU15 treatments, and yet no significant differences were observed between the LCRU and LCRU15 treatments. Notably, the nitrogen used efficiency of choy sum increased to 68.90% with the LCRU15 treatment, compared to 64.29% with the LCRU treatment. The levels of soil available nitrogen, NO_3_^−^−N, and NH_4_^+^−N were augmented by the LCRU and LCRU15 treatments. Meanwhile, soil urease and nitrate reductase activities were increased by 22.4%-28.6% and 12.3%-14.5%, respectively. Moreover, soil high-throughput sequencing results illustrated that the LCRU15 treatment enhanced the diversity and abundance of bacteria, particularly the abundance of Actinobacteria, Firmicutes, and Cyanobacteria, which can accelerate the decomposition of organic matter. In short, LCRU improves choy sum yield by influencing soil properties, enzyme activity, and microbial communities. These findings are anticipated to offer practical value for the sustainable application of LCRU in agriculture.

## 1 Introduction

In agricultural production, enhancing the efficiency of fertilizer use has emerged as a major challenge (Melino et al., 2022). In particular, the traditional mineral nitrogen fertilizers (such as urea) are easily leached from the soil, approximately 40–70% of nitrogen is lost through various channels (Zhang et al., 2021; Chen et al., 2018), including nitrate leaching, runoff, and erosion, ammonia (NH_3_) volatilization, and gas emissions (Abalos et al., 2022; Verma et al., 2023). Consequently, the inefficient use of nitrogen fertilizer causes environmental pollution and a substantial waste of resources. To tackle these challenges, the development of slow/controlled-release nitrogen fertilizers, specifically the formulation of coated fertilizers, has been recognized as a promising approach to enhancing nutrient efficiency and minimizing environmental impacts (Channab et al., 2023; Govil et al., 2024). Controlled-release fertilizers (CRFs) are specifically engineered to control the rate at which nutrients are released, aligning nutrient supply with the real-time needs of the crops. Compared with traditional fertilizers, CRF gradually releases nutrients, regulating crop nutrient absorption, soil physicochemical properties, as well as enzyme activity, thus positively impacting crop yield. Moreover, numerous studies have documented the advantages of CRF, highlighting benefits including reducing labour costs, lowering the risk of seedling burn, promoting root development, nutrient absorption, and enhancing crop yield and quality (Vejan et al., 2021; Yang et al., 2015; Miyatake et al., 2019; Tian et al., 2016). Hence, CRF presents a promising approach for advancing sustainable agricultural practices. Nonetheless, in numerous studies, the coating materials typically utilized in CRF are sourced from petroleum-based raw materials. These materials are expensive, non-renewable and non-biodegradable, leading to secondary environmental pollution and hindering the development of modern green agriculture. As a result, renewable and biodegradable coating materials are essential as substrates for CRF.

Recent scholarly focus has shifted towards the development of biomass-based raw materials for creating environmentally friendly and biodegradable CRFs (Fertahi et al., 2021; Perez and Francois, 2016; Tian et al., 2021; Zhong et al., 2013). Various alternatives such as vegetable oil (Paraskar et al., 2021), chitosan (Frank et al., 2020), starch (Li H. et al., 2019; Tian et al., 2022), lignin, and cellulose (Riseh et al., 2023; Huber et al., 2012) have been investigated. Among these materials, lignin is notable for its renewability, cost-effectiveness, and natural biodegradability (Fertahi et al., 2021). Besides, industrial lignin, primarily obtained as a by-product from the pulp and paper industry, as well as from cellulosic ethanol and other biorefinery processes (Bajwa et al., 2019), presents opportunities for reducing production costs and creates a pathway for waste valorisation. While the application of lignin in CRFs has been initially explored in laboratory settings, there are no field studies documenting the use of lignin-based CRFs on crops have yet been published. Furthermore, soil microbes play an essential role in regulating critical soil biological processes, including nutrient cycling and material and energy conversion. Additionally, fertilization has substantial effects on the soil microbial community structure, as changes in soil chemical composition initiated by nutrient release from fertilizers result in changes to the diversity and relative abundance of soil microbial communities (Zhang et al., 2018; Iqbal et al., 2022). Furthermore, soil enzyme activities are indirectly affected by the soil microbial communities associated with distinct types of fertilizers, thereby affecting the concentrations of carbohydrates, amino acids, carboxylic acids, starch, and cellulose (Li et al., 2023). Nonetheless, the impact of lignin-based controlled-release nitrogen fertilizers (LCRNFs) on the diversity of soil microbial communities remains unexplored. Thus, it is essential to explore how soil microbial communities in crop-growing systems are regulated by LCRNFs. In early potted studies, it was determined that LCRNFs and a 15% reduction in fertilizer content could promote choy sum growth and nitrogen use efficiency (Chen et al., 2023). In this study, the effects of LCRNFs on the growth, quality and yield of choy sum were examined in greater detail through two-season field experiments. Simultaneously, assessments were conducted to evaluate how lignin-based coated fertilizers influence soil physicochemical properties and enzyme activities in late-season choy sum. Additionally, the study explored the soil bacterial community structure under the lignin-based coated fertilizer and crop growth system. The relative contributions of soil nutrient supply, carbon cycle enzymes, and nitrogen cycle enzymes to the diversity of bacterial communities were also examined. These findings seek to establish a theoretical foundation for the application of biomass-based slow and controlled-release fertilizers in agricultural practices.

This study aims to investigate the effects of lignin-based controlled-release urea (LCRU) on the quality enhancement and efficiency improvement of choy sum. Besides, the study examines the interactions between LCRU, soil physicochemical properties, and soil bacterial diversity, especially focusing on the interpretation between nitrogen nutrient supply and soil nitrogen-converting enzyme activity. The findings are designed to offer valuable insights into the application of biomass-based slow and controlled-release fertilizers in crop cultivation.

## 2 Materials and methods

### 2.1 Experimental design and field management

The experimental site was selected in Zhucun, Zengcheng District, Guangzhou, Guangdong Province (113.695°E, 36.276°N), which has a subtropical monsoon climate. Moreover, the annual rainfall in the Zhucun area for the years 2021, 2022, and 2023 was 1644.5, 1737.3, and 1787.3 mm, respectively, with average temperatures of 21.5, 21.8, and 22.8°C. The vegetable garden soil is used, with its properties detailed in **Table 1**. Furthermore, the seedling period was from September 9 to October 10, 2021, and the first season field experiment was conducted from October 10, 2021, to January 11, 2022. Additionally, the second seedling period was from October 7 to November 8, 2022, and the second season field experiment was conducted from November 9, 2022, to February 9, 2023.

**Table 1:** Physical and chemical properties of soil.

Time (years)	pH	Organic matter	Total nitrogen	Available nitrogen	Available phosphorus	Available potassium
		(g/kg)	(g/kg)	(mg/kg)	(mg/kg)	(mg/kg)
2021	6.73	17.7	1.23	113.53	21.43	75.80
2022	6.72	18.0	1.40	121.12	23.88	82.60

The experimental plot covered an area of 48 m² (6 m in length and 8 m in width), with each subplot measuring 8 m in length and 1.2 m in width, and a furrow width of 0.3 m. The four treatments were established using a single-factor randomized block design and were designated as follows: no fertilization (CK), conventional urea (CU, 150 kg N/hm²) divided twice per season, previous research has illustrated that a 15% reduction in urea content has a lesser effect on plant growth compared to a 30% reduction, and consequently design two test groups using LCRU (44% N, 150 kg N/hm²) and LCRU15 (127.5 kg N/hm²) apply once per season. Meanwhile, phosphorus and potassium were uniformly applied across all treatments in the form of superphosphate (91.67 kg P_2_O_5_/hm²) and potassium chloride (195.24 kg K_2_O/hm²), respectively, as basal applications. All other agronomic practices remain unchanged.

Choy sum seedlings were pre-cultivated and transplanted at the three-leaf stage, following a planting density of 40×40 cm. During the late growth stage of choy sum, regular pesticide applications were carried out to manage pests and diseases. Harvesting took place once the choy sum reached a compact growth stage. Yield assessments were limited to the three central rows of each plot, and soil samples were collected from the same central rows for further analysis. Following the first season of the choy sum experiment, the farmers planted a crop of sweet corn to optimize the use of arable land. The corn was sown directly without applying any base fertilizer, and topdressing was only applied during the jointing and grain-filling stages, primarily employing compound fertilizer (90 kg N/hm², 90 kg P_2_O_5_/hm², 90 kg K_2_O/hm²), and the cultivation cycle lasted 80 days. Prior to the second season of the choy sum experiment, the land was plowed and thoroughly mixed to minimize soil fertility variations resulting from the fertilization treatments applied during the first season.

### 2.2 Sampling and chemical analyses

To evaluate the impact of LCRU on soil nutrient availability and physicochemical properties, numerous parameters were measured, including soil available nitrogen content, NO_3_^−^, NH_4_^+^, organic matter, pH, urease, and nitrate reductase activity. Besides, soil samples were gathered from a depth of 0–20 cm using specialized soil drilling tools, and each collected soil sample was subsequently divided into three subsamples. An additional retained sample was transported to the laboratory and preserved at -80°C for DNA extraction. Subsequently, a 2 g aliquot of soil intended for the determination of available nitrogen content was placed in a 250 mL glass bottle containing 100 mL of 0.01 mol·L^−^¹ CaCl_2_ solution and agitated at 250 rpm for 1 hour. Concentrations of NH_4_^+^-N and NO_3_^−^-N were quantified utilizing a continuous flow analyser (CFA, AMD France). Meanwhile, soil pH was assessed by employing a pH meter with a 1:2.5 soil-to-water ratio. The activities of soil enzymes, such as urease and nitrate reductase, were measured using an ELISA kit supplied by Shanghai Heng yuan Biological Technology Co. Ltd. (Shanghai, China). Upon the harvest of the choy sum, the produce was immediately transported to the laboratory for quality assessment. Furthermore, metrics including vitamin C content, soluble sugar, and soluble protein were evaluated. Plant dry biomass was determined by oven-drying the samples at 75°C upon harvest. Additionally, key variables including plant height, stem thickness, SPAD value, above-ground dry weight, subterranean dry weight, nitrogen uptake, as well as nitrogen use efficiency were meticulously evaluated and calculated. The nitrogen content of dried plant samples was determined by the Kjeldahl method after digestion.

Vitamin C was performed by using the 2, 6-dichlorophenol indophenol titration method (Shyamala and Jamuna, 2010). 0.5 g fresh leaves were ground into pulp with 3 mL 1% oxalic acid, 1 mL 30% zinc sulfate and 1 mL 15% potassium ferrocyanide. 10 mL extracting solution was mixed with 1 mL phosphate-acetic acid, 2 mL 5% vitriol and 4 mL ammonium molybdate. After 15 min, the mixed solution was determined at 500 nm by UV-VIS spectrophotometer (Shimadzu UV-16A, Shimadzu, Japan).

Soluble sugar content was performed by anthronesulfuric acid colorimetry method (Song et al., 2012). 0.5 g fresh leaves were heated on boiling water bath with 10 mL distilled water for 30 min. 0.1 mL supernatant was mixed with 1.9 mL distilled water, 0.5 mL anthrone ethyl acetate and 5 mL vitriol. After shaking, the soluble sugar was detected by UV-VIS spectrophotometer (Shimadzu UV-16A, Shimadzu, Japan) at 630 nm.

Soluble protein content in lettuce was examined by Coomassie brilliant blue G-250 dye method. A total of 0.5 g fresh lettuces was ground into pulp by liquid nitrogen with 5 mL distilled water. The extract solution was centrifuged at 10000 rpm for 10 min at 4°C, and 0.05 mL supernatant was combined with 0.95 mL distilled water and 5 mL Coomassie brilliant blue G-250 solution (Sigma, USA, 0.1 g / L). After 2 min, the soluble protein content was detected at 595 nm by UV-spectrophotometer (Shimadzu UV-16A, Shimadzu, Japan).

### 2.3 Soil bacterial diversity analysis

In accordance with the manufacturer’s guidelines, DNA samples were extracted from 12 different soil samples using the DNeasy Powersoil Kit (Qiagen, Hilden, Germany). The absorbance ratios (A260/A280) of these DNA samples ranged between 2.0 and 3.0. Subsequent high-throughput sequencing of bacterial communities was executed on the Illumina NovaSeq 6000 platform by Majorbio Bio-pharm Technology Co., Ltd. (Shanghai, China). Amplification of the V4 region of the bacterial 16S rRNA gene was performed using primers 515F and 806R. Data denoising for specific 16S/ITS regions was conducted on the Qiime 2 platform utilizing the DADA2 algorithm. Amplicon Sequence Variants (ASVs) for each sample were thereby obtained. Taxonomic classification of bacterial identities was performed using the Silva 138 and Unite 8.0 databases, through a Bayes taxonomy classifier. To standardize sequencing depth for downstream analyses, the ASV table was rarefied to the minimum sequence numbers observed across all samples.

Quality filtering yielded a total of 86,960 high-quality 16S rRNA sequences from 14 soil samples, resulting in 19,850 bacterial. Various alpha diversity indices, including Sobs, Chao 1 estimator, Shannon index, and Shannoneven index, were calculated utilizing the Qiime 2 platform. Principal Component Analysis (PCA) based on the Bray-Curtis dissimilarity metric was employed to discern overall differences in microbial diversity structure between CK and treatments. Biomarkers for each treatment were identified using the Linear Discriminant Analysis Effect Size method on the Galaxy Platform. Furthermore, annotation of the bacterial community**’**s functional profile was conducted using FAPROTAX on the online Majorbio Cloud Platform.

### 2.4 Statistical examinations

Microsoft Excel 365 and SPSS 22.0 were used for data organization and analysis. The results were expressed as mean ± standard error. Duncan**’**s multiple comparison tests (*p* < 0.05) was used, and data denoted by the same letters indicate insignificant differences between the means. Origin 2022 software was used for plotting.

## 3 Results

### 3.1 Effects of different fertilization treatments on the growth of choy sum

**Figure 1:**
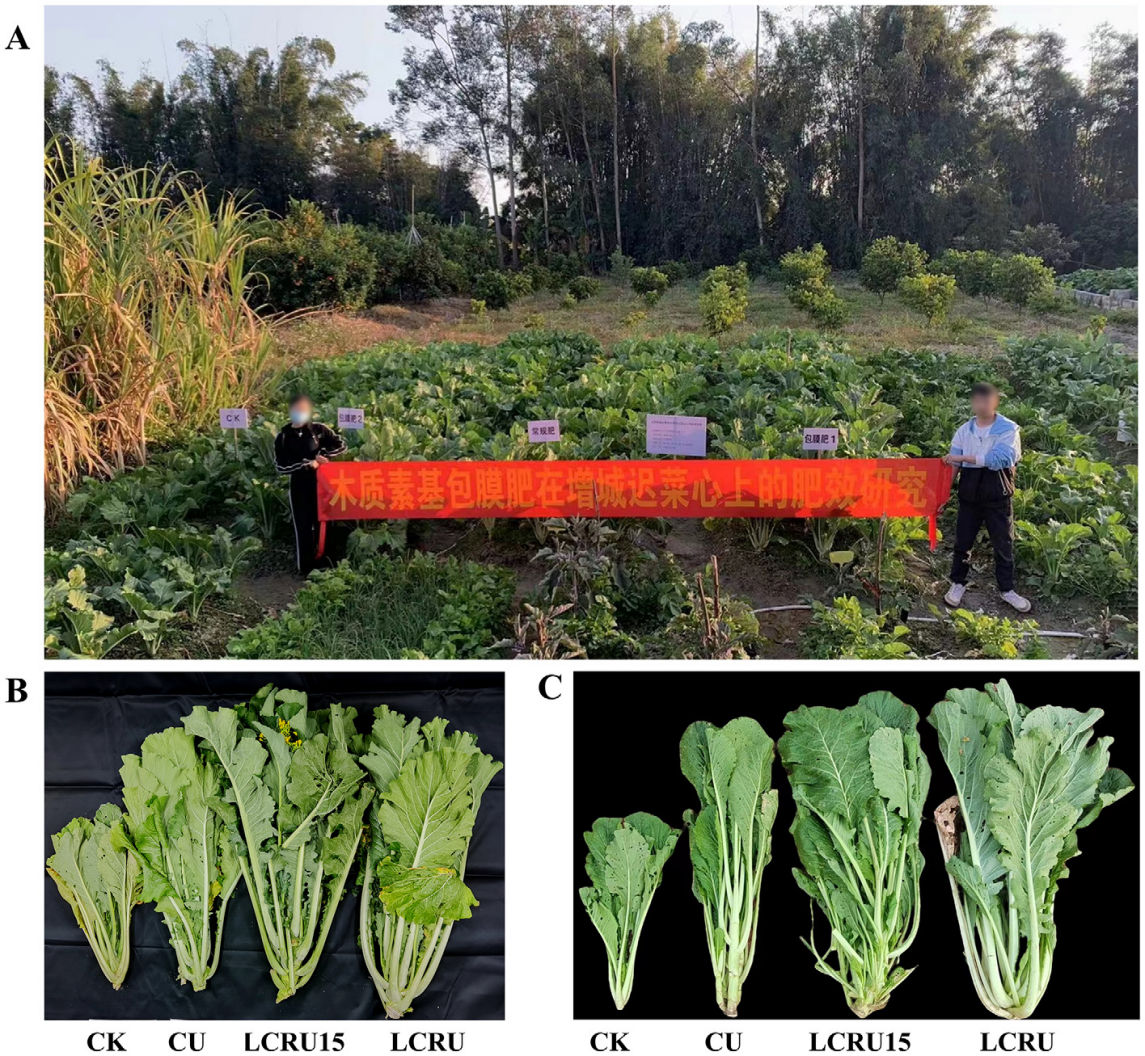
Scene map of the choy sum test site **(A)**, choy sum pictures in 2021 **(B)** and 2022 **(C)**.

In agricultural production, the rational application of fertilizers plays a decisive role in crop growth. As shown in **Table 2**, throughout the two-season field experiment, the fertilization treatments notably enhanced the growth metrics of choy sum than the CK (**Figure 1**). Specifically, the biennial mean values for plant height, stem diameter, SPAD, as well as above-ground dry weight for the LCRU treatment were 80.36 cm, 4.92 mm, 45.75, and 7482.59 kg/hm², respectively. These metrics were substantially increased by 40.27%, 26.97%, 52.02%, and 38.62%. The mean values for plant height, stem diameter, SPAD, and above-ground dry weight under the LCRU treatment were modestly superior to those observed under the LCRU15 treatment. Nonetheless, the LCRU15 treatment revealed increments of 24.76%, 26.97%, 43.23%, and 30.86% in plant height, stem diameter, SPAD, and above-ground dry weight, respectively, compared with the CU treatment. It is essential to note that during the 2022 growing season, plant height, stem diameter, SPAD readings, and above-ground dry weight were greater across all treatments than the previous year. This increase may be attributed to the inherent soil fertility.

**Table 2:** Effects of different fertilization treatments on the growth indicators of choy sum in field.

Time (years)	Treatment	Plant height (cm)	Stem diameter(mm)	SPAD	Above-ground dry weight(kg/hm^2^)
2021	CK	34.91±1.16d	1.90±0.21c	35.57±1.92c	2363.89±80.92d
	CU	51.17±1.59c	3.52±0.14b	40.37±181b	5018.99±223.49c
	LCRU	73.79±1.67a	4.67±0.22a	46.33±2.09a	6753.46±125.72a
	LCRU15	65.33±2.40b	4.15±0.18a	49.13±1.25a	6243.34±114.81b
2022	CK	42.12±1.64c	2.58±0.07c	31.24±1.62c	2715.08±116.15c
	CU	63.41±0.90b	4.23±0.20b	40.98±1.80b	5777.35±190.04b
	LCRU	86.93±1.76a	5.17±0.23a	45.17±1.34a	8211.71±220.72a
	LCRU15	77.62±1.67a	5.04±0.25a	47.82±1.47a	7883.85±190.04a

Data in the table indicate mean ± standard deviation (n=5). Different letters of the same index treated in different ways indicate significant differences (*p*<0.05), the same below.

Different letters indicate significant differences between different treatments on the same day (*p*<0.05), and the same below.

### 3.2 Effects of different fertilization treatments on the quality of choy sum

**Figure 2:**
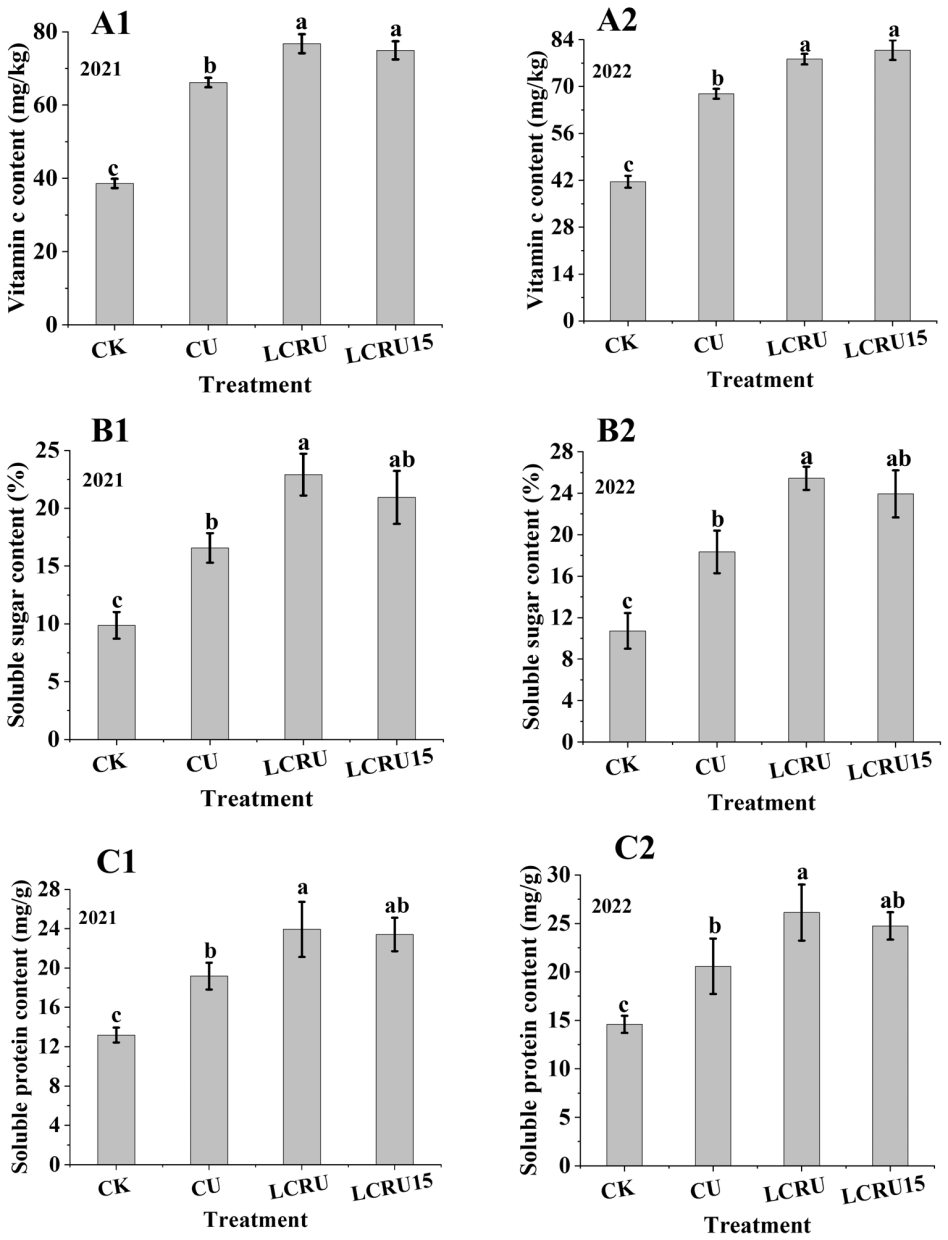
Effects of LCRU application on vitamin C (2021: **A1**, 2022: **A2**), soluble sugar (2021: **B1**, 2022: **B2**) and soluble protein (2021: **C1**, 2022: **C2**) concentrations in choy sum. The different lowercase letters indicate significant differences among treatments (p<0.05).

The palatability of choy sum is closely associated with its quality, making it a crucial metric for evaluation. The field experiment revealed that the levels of vitamin C, soluble sugar, and soluble protein in choy sum content to fertilization treatments were substantially elevated than the CK treatment (**Figure 2**). Besides, the concentrations of vitamin C, soluble sugar, and soluble protein in choy sum treated with LCRU during the two-season field experiment were significantly higher than those treated with CU. Specifically, under the LCRU treatment in the bi-seasonal field study, the choy sum exhibited concentrations of 78.81 mg/100g for vitamin C (**Figures 2A1, A2**), 24.17% for soluble sugar (**Figures 2B1, B2**), and 25.03 mg/g for soluble protein (**Figures 2C1, C2**). These concentrations demonstrate significant increases of 17.64%, 38.50%, and 25.88%, respectively, compared to the CU treatment. It is essential to note that no significant differences were observed in the levels of vitamin C, soluble sugar, and soluble protein between choy sum treated with LCRU and LCRU15 during the field experiment. In summation, the quality of the choy sum is positively affected by the application of LCRU, and a 15% reduction in urea does not significantly reduce this improvement in quality.

### 3.3 Effects of LCRU on element accumulation and N use efficiency of choy sum

**Figure 3:**
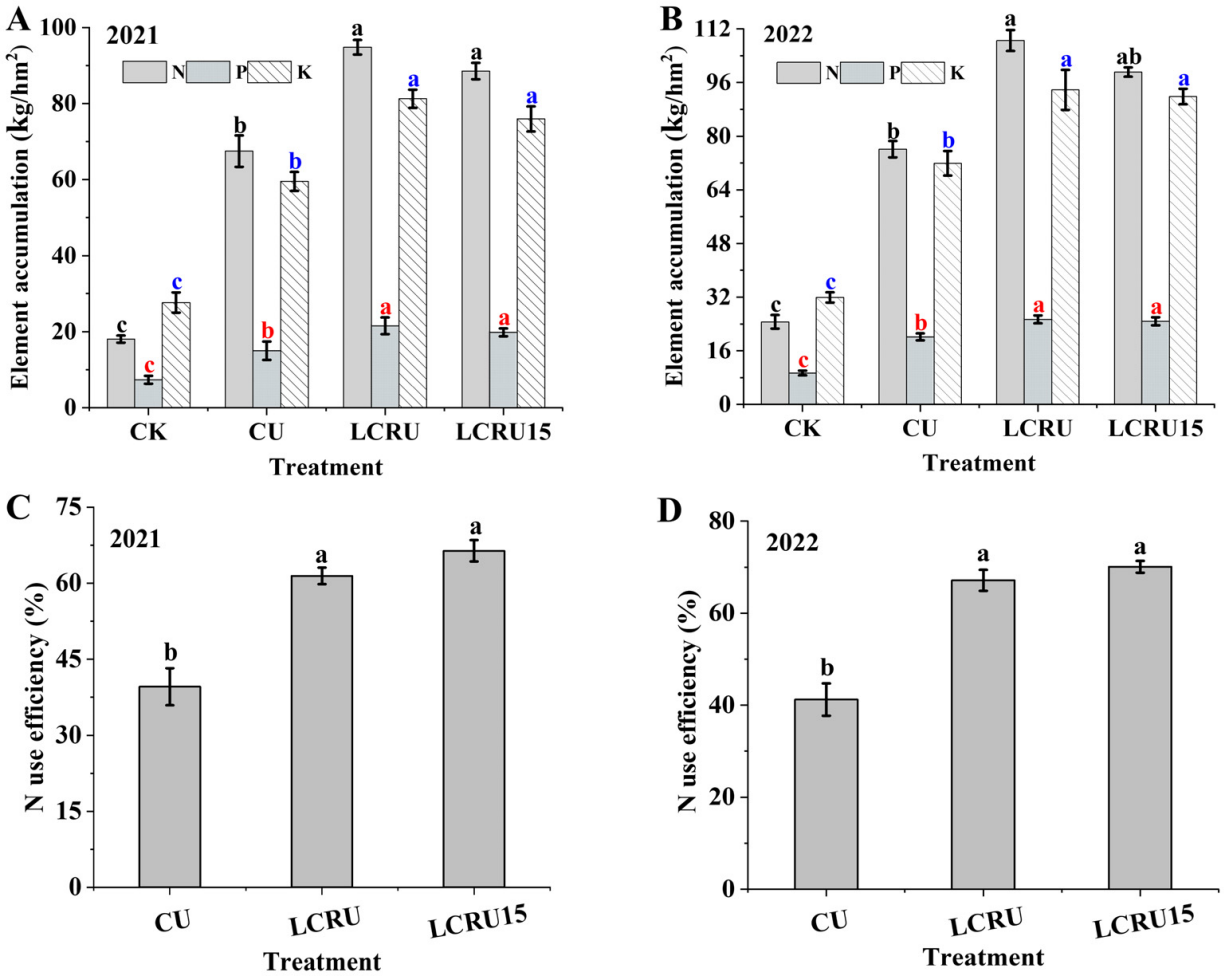
Effects of LCRU application on choy sum element accumulation (2021: **A**, 2022: **B**), and N use efficiency (2021: **C**, 2022: **D**). The different lowercase letters indicate significant differences among treatments (p<0.05).

There were no significant differences were determined in the absorption of N, P, and K by choy sum between the LCRU and LCRU15 treatments (**Figures 3A, B**). Notably, regarding the accumulation of N, P, and K in field-grown choy sum, the average accumulations for the LCRU treatments over two years were recorded as 101.67 kg/hm², 23.45 kg/hm², and 87.54 kg/hm², respectively. Compared with the CU treatment (71.82 kg/hm², 17.56 kg/hm², and 65.70 kg/hm²), the LCRU treatments demonstrated increases of 41.56%, 33.54%, and 33.24%. Furthermore, the two-year accumulation of P and K in fields under both LCRU and LCRU15 treatments exhibited negligible variance. In summary, a 15% reduction in the dosage of lignin-coated fertilizer, while maintaining stable absorption levels of nitrogen, phosphorus, and potassium in choy sum, effectively satisfies the plant**’**s nutritional requirements. The efficiency of N fertilizer uses efficiency as a metric to gauge how well crops exploit the available nitrogen within a specific timeframe. In the two-season field experiment, the LCRU15 treatment demonstrated an average nitrogen use efficiency of 68.24%, which was 68.90% higher than efficiency of 40.40% of the CU treatment (**Figures 3C, D**). Moreover, the mean nitrogen use efficiency under the LCRU15 treatment surpassed that of the LCRU regimen (64.29%), implying that a 15% reduction in the lignin-based coated fertilizer could substantially enhance nitrogen use efficiency for the choy sum at season.

### 3.4 Effects of LCRU application on soil N content

**Figure 4:**
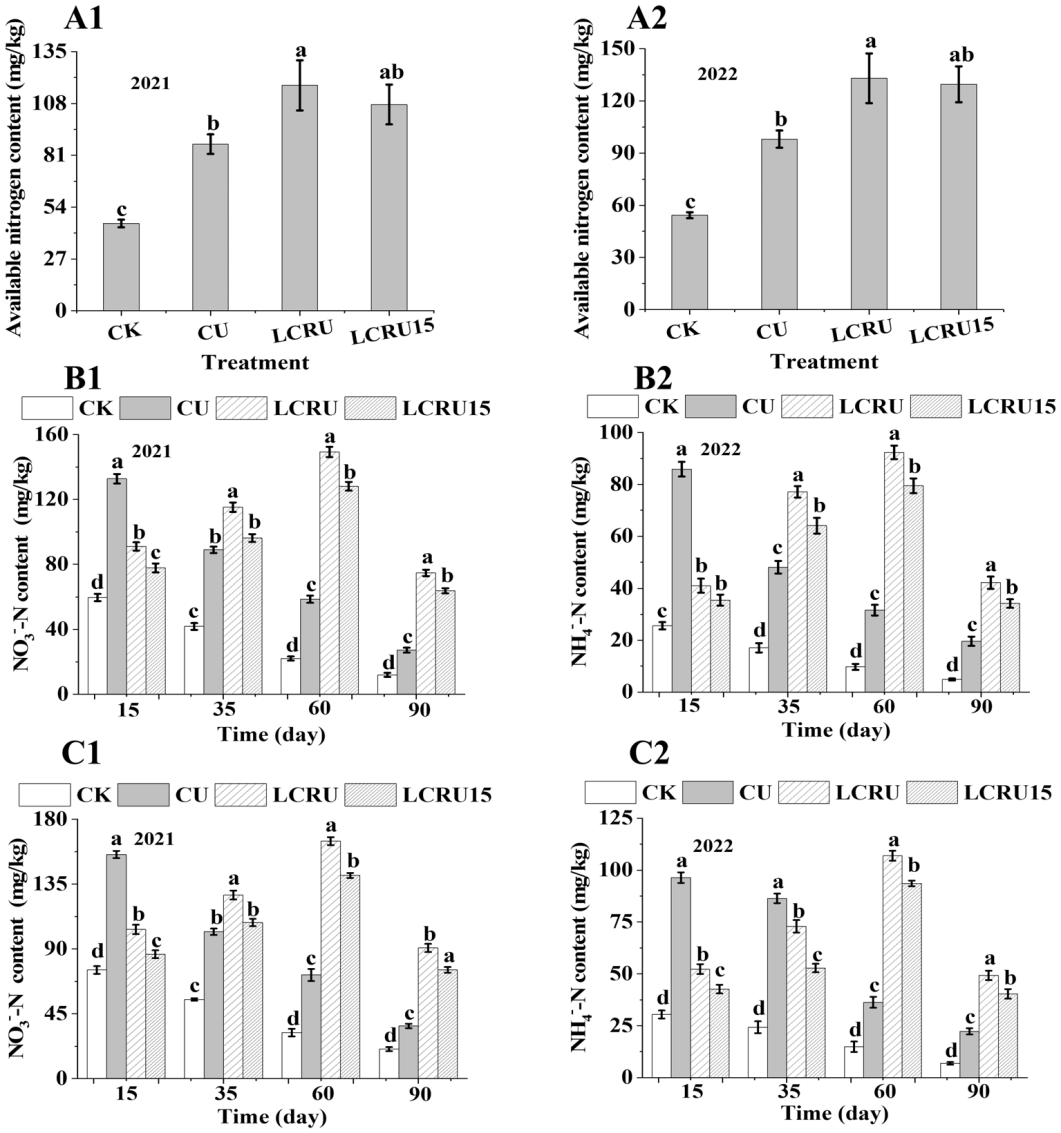
Effects of LCRU application on soil nitrogen content (2021: **A1**, 2022: **A2**), NO_3_^−^-N (2021: **B1**, 2022: **B2**) and NH_4_^+^-N (2021: **C1**, 2022: **C2**). The different lowercase letters indicate significant differences among treatments (p<0.05).

For the purpose of assessing the nitrogen supply capacity within the soil, measurements were taken of available nitrogen content, NO_3_^−^-N, and NH_4_^+^-N levels. Compared to the CK treatment, a significant increase in available nitrogen content was noted in the choy sum that received fertilization (**Figures 4A1, A2**). It is worth noting that, in comparison to the available nitrogen content in the soil before fertilization (113.53 mg/kg in the first season), merely the LCRU treatment indicated a 3.43% increase, whereas all other fertilization treatments resulted in a decrease. Following the second season of choy sum harvest, the available nitrogen content increased by 9.8% and 6.9% for LCRU and LCRU-treated plots, respectively, compared to the levels before the experiment. In the field study, the average available nitrogen content in the soil for choy sum under the LCRU treatment was 125.21 mg/kg, marking a significant 35.51% increase than the CU treatment, which had an available nitrogen content of 92.40 mg/kg. Despite the fact that the LCRU treatment was marginally superior to the LCRU15 treatment, which had an average soil available nitrogen content of 118.47 mg/kg, both LCRU and LCRU15 increased the soil’s available nitrogen content. During the field experiments, fluctuations in soil NO_3_^−^-N (**Figures 4B1, B2**) and NH_4_^+^-N (**Figures 4C1, C2**) contents within the root zone were documented, varying according to the different growth stages and treatments. For the two-season field CU treatments, peaks in NO_3_^−^-N and NH_4_^+^-N content were recorded on the 15th day, averaging 91.13 and 144.10 mg/kg, respectively. Upon the 15th day, a decline in both NO_3_^−^-N and NH_4_^+^-N contents was observed for the CU treatments as time progressed. Notably, the NO_3_^−^-N content for the LCRU treatment initially increased (from 97.25 to 157.03 mg/kg) prior to declining (from 157.03 to 82.64 mg/kg) over time. A consistent trend in NO_3_^−^-N content over time was observed for both LCRU and LCRU15 treatments. Additionally, the peak NO_3_^−^-N content for the LCRU treatment over both seasons was 8.97% higher than that of the CU treatment, whereas the distinctions between LCRU15 and CU treatments was relatively minor. Similar patterns were observed for changes in NO_3_^−^-N and NH_4_^+^-N contents across all treatments during the two-season field study. These findings indicate that the introduction of lignin-based coated fertilizer positively affects the levels of NH_4_^+^-N and NO_3_^−^-N in the soil, thereby enhancing nitrogen uptake by crops. Moreover, even with a 15% reduction, soils treated with lignin-based coated fertilizer maintained high NH_4_^+^-N and NO_3_^−^-N content over an extended period, indicating that the decreased fertilizer application did not undermine its effectiveness.

### 3.5 Effect of LCRU application on soil properties

**Figure 5:**
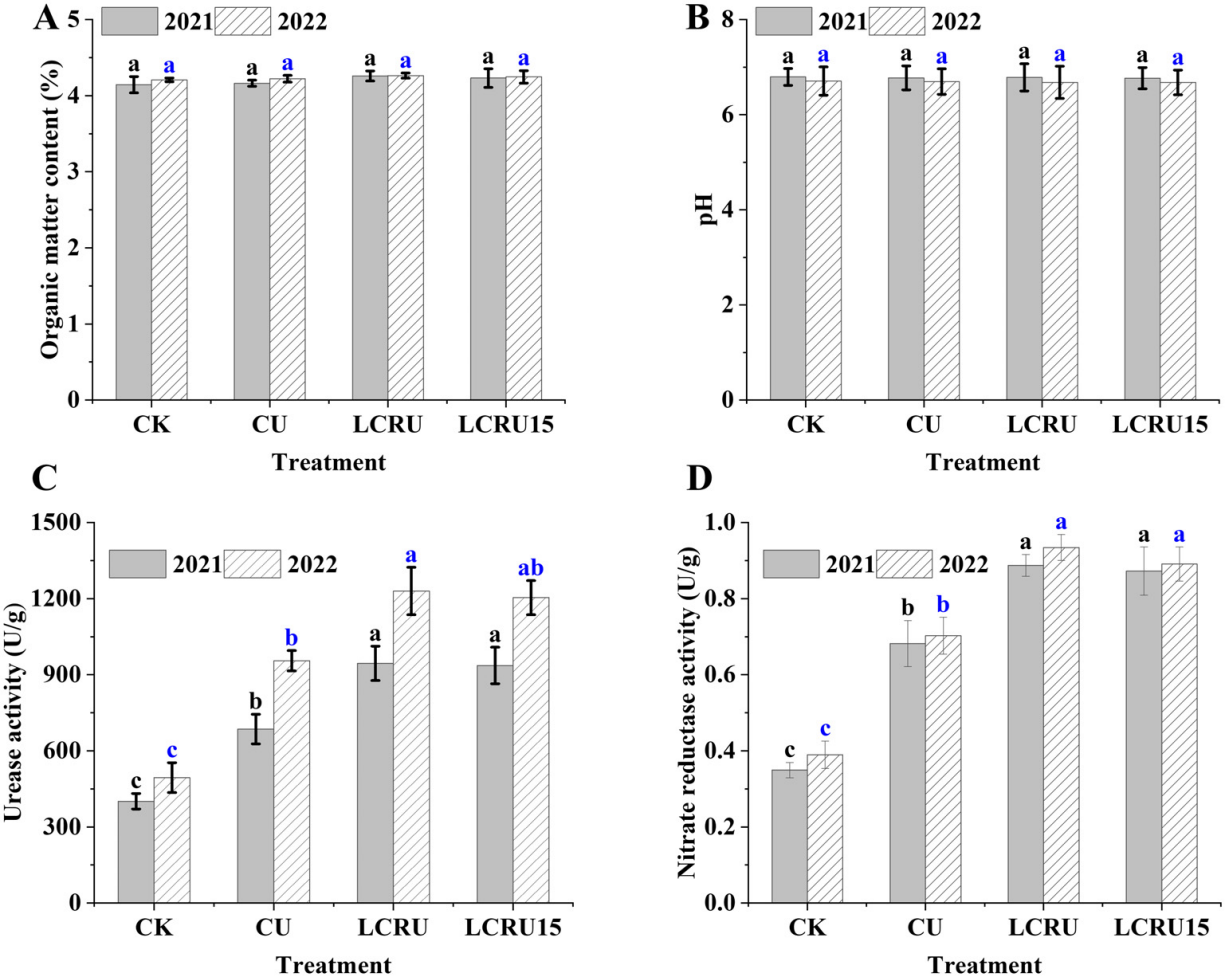
Effects of LCRU application on soil organic matter (2021: **A**, 2022: **B**), pH, urease and nitrate reductase activity (2021: **C**, 2022: **D**). The different lowercase letters indicate significant differences among treatments (p<0.05).

As shown in the **Figures 5A, B**, no significant distinctions were observed in the effects of various fertilization treatments on soil organic matter and pH in the late choy sum. Moreover, soil urease and nitrate reductase were evaluated. As illustrated in **Figures 5C, D**, during the two-season field experiments, the activities of soil urease and nitrate reductase in the LCRU treatment were 1087.37 U/g and 0.91 U/g, respectively, with no significant difference compared to the LCRU15 treatment (1070.04 and 0.88 U/g). Nonetheless, the average activities of soil urease and nitrate reductase in the LCRU treatment were significantly higher than those in the CU treatment (820.40 and 0.69 U/g), with increases of 32.54% and 31.58%, respectively. These findings indicate that both the full dose of lignin-based coated fertilizer and a 15% reduction can substantially increase soil urease and nitrate reductase activities.

### 3.6 The response of soil bacterial diversity to LCRU

**Figure 6:**
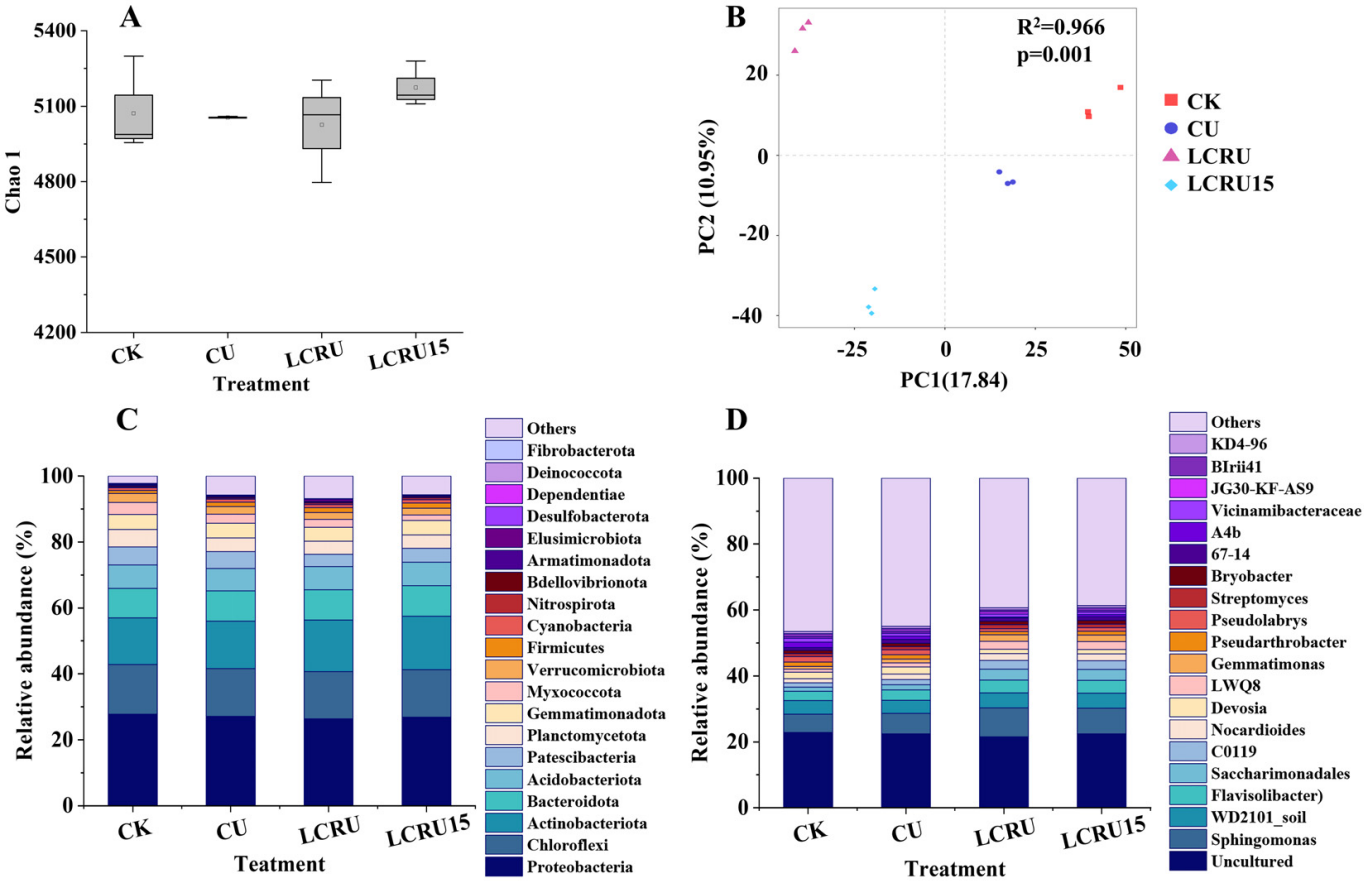
Effects of LCRU application on Chao1 **(A)**, PC2 **(B)**, rhizosphere bacteria phylum **(C)** and genera **(D)** levels.

It is illustrated that bacterial abundance and diversity were significantly affected by the fertilization treatments (**Figure 6**). In particular, the Chao1 index of soil bacteria increased by 17.95% and 15.54% for the LCRU15 treatment in comparison to the CU and LCRU treatments, respectively (**Figure 6A**). In contrast, the LCRU treatment revealed only a marginal increase of 0.23% over the CU treatment. To identify compositional differences among the treatments, Principal Coordinates Analysis (PCoA), a non-parametric dimensionality reduction technique, was applied at the Amplicon Sequence Variant (ASV) level. A statistically significant distinction among the treatments was detected along the first principal component (**Figure 6B**, R² =0.966, *p*= 0.001). The Unweighted-Unifrac distance matrix accounted for 17.84% of the variance in the composition of bacterial and fungal communities along the first principal component, indicating substantial variability across treatments. Moreover, to assess the impact of LCRU on the bacterial community, high-throughput 16S rRNA amplicon sequencing was conducted on soil samples. Except for that, **Figures 6A, B** demonstrates the composition and relative abundance of bacterial phyla under different treatment conditions. The dominant bacterial phyla in the soil samples were Proteobacteria (26.33-27.81%), Chloroflexi (14.33-15.07%), Actinobacteriota (14.06-16.22%), Bacteroidota (9.00-9.29%), Acidobacteriota (6.78-7.09%), Patescibacteria (6.78-7.09%), Planctomycetota (3.77-5.44%), and Gemmatimonadota (4.16-5.33%), among others. These phyla collectively accounted for over 93.20% of bacteria in the respective treatments. Concerning bacterial genera, the most dominant was Uncultured (20.54-22.89%), Sphingomonas (5.5-8.79%), WD2101_soil_group (3.25-4.53%), Flavisolibacter (2.77-3.86%), Saccharimonadales (1.24-3.31%), C0119 (1.35-2.71%), Nocardioides (1.28-2.07%), Devosia (1.99-2.16%), and LWQ8 (0.87-2.37%), which together constituted more than 53.46% of the bacteria in each respective treatment. Compared with the CK treatment, the fertilization treatments induced significant changes in both the composition and abundance of dominant bacterial phyla and genera. Specifically, among the top 20 phyla and genera exhibiting the highest bacterial abundance in LCRU and LCRU15 treatments, increases were observed in ten phyla, including Proteobacteria, Actinobacteriota, Bacteroidota, Patescibacteria, Firmicutes, Cyanobacteria, Nitrospirota, and Bdellovibrionota, as well as in ten genera including Sphingomonas, WD2101_soil_group, Flavisolibacter, Saccharimonadales, C0119, Nocardioides, and Streptomyces. In summary, the application of LCRU significantly influenced bacterial abundance in the soil, and this effect was further magnified when the usage of LCRU was reduced by 15%. Hence, it can be inferred that the judicious application of LCRU has the potential to positively modulate soil bacterial diversity and abundance.

### 3.7 The relationship between bacterial diversity and LCRU soil environmental factors

**Figure 7:**
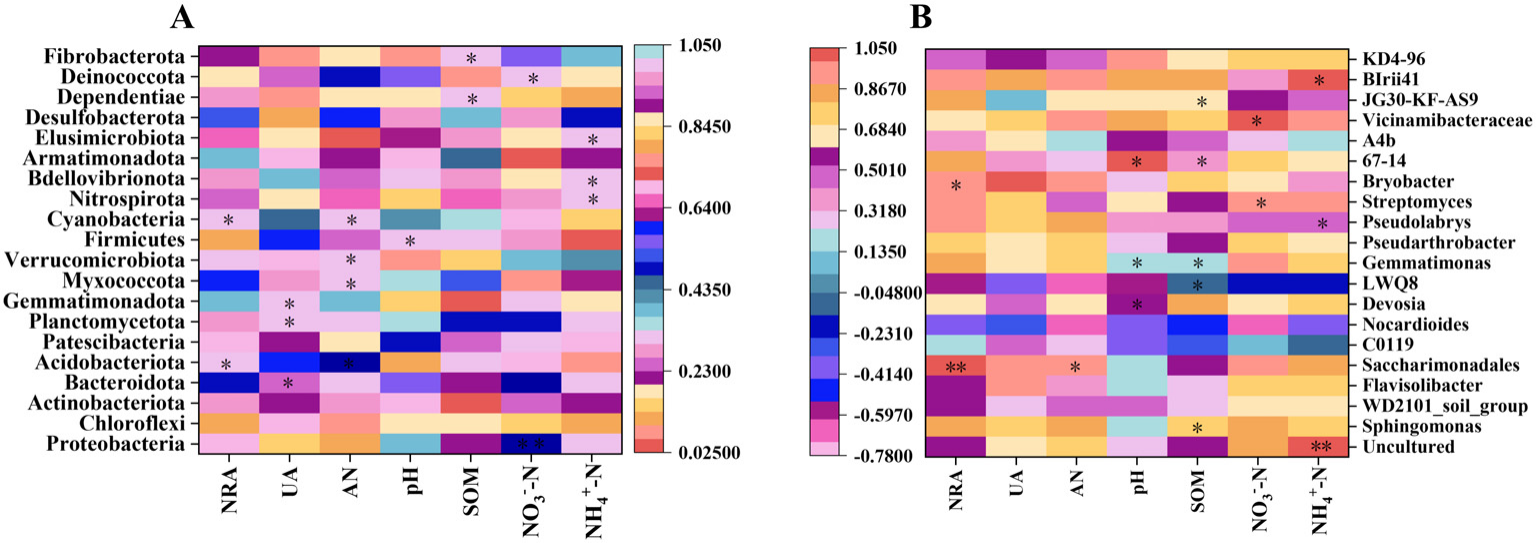
The correlation between soil rhizosphere bacterial phyla **(A)** and genera **(B)**, and various soil environmental indicators. Stars denote significance at *p*<0.05 and *p*<0.01 probability levels (* and **, respectively).

An intricate relationship between bacterial communities in soil and various soil environmental indicators, such as nitrate reductase activity (NRA), urease activity (UA), available nitrogen (AN), pH, soil organic matter (SOM), NO_3_^−^-N, and NH_4_^+^-N, has been observed (**Figure 7**). Meanwhile, these factors collectively influence the composition, diversity, and functional dynamics of soil bacterial communities. The study demonstrated significant correlations between bacterial phyla related to the nitrogen cycle and these soil indicators. For instance, significant correlations were established between NRA and both Acidobacteriota and Cyanobacteria (*p*<0.05). Similarly, Actinobacteriota and Gemmatimonadota indicated significant correlations with UA (*p*<0.05). Furthermore, a substantial positive correlation was found between AN and multiple bacterial phyla, including Acidobacteriota, Planctomycetota, Myxococcota, Verrucomicrobiota, and Cyanobacteria (*p*<0.05). Noteworthy correlations were also observed between NO_3_^−^-N and the phyla Proteobacteria, Bacteroidota, Patescibacteria, and Deinococcota (*p*<0.05), as well as between NH_4_^+^-N and the phyla Nitrospirota, Bdellovibrionota, and Elusimicrobiota. At the genus level, both positive and negative correlations were observed between bacterial genera and soil environmental indicators (**Figure 7B**). For instance, a significant positive correlation was identified between the genus Saccharimonadales and NRA (*p*<0.05). Conversely, UA was substantially negatively correlated with the genus LWQ8 but positively correlated with Bryobacter (*p*<0.05). AN was significantly positively correlated with Saccharimonadales (*p*<0.05). Additionally, significant positive correlations were identified between NO_3_^−^-N and genera like Streptomyces and Vicinamibacteraceae (*p*<0.05), as well as between NH_4_^+^-N and genera including Pseudolabrys and BIrii41 (*p*<0.05). SOM indicated significant correlations with Sphingomonas, Gemmatimonas, 67-14, and JG30-KF-AS9, while pH correlated significantly with Devosia, Gemmatimonas, Pseudarthrobacter, and 67-14 (*p*<0.05). In summary, the findings indicate that the implementation of the LCRU regimen is associated with the enhancement of bacterial phyla related to the nitrogen cycle, including Acidobacteriota, Cyanobacteria, Actinobacteriota, Gemmatimonadota, Planctomycetota, Myxococcota, Verrucomicrobiota, Nitrospirota, Bdellovibrionota, and Elusimicrobiota. Additionally, specific genera, including Saccharimonadales, Bryobacter, Streptomyces, Vicinamibacteraceae, Pseudolabrys, and BIrii41, were likewise positively influenced, emphasizing the potential of LCRU to improve soil microbial diversity associated with nutrient cycling and soil health.

### 3.8 LCRU regulates soil properties, enzyme activity, and bacterial diversity to affect choy sum yield

**Figure 8:**
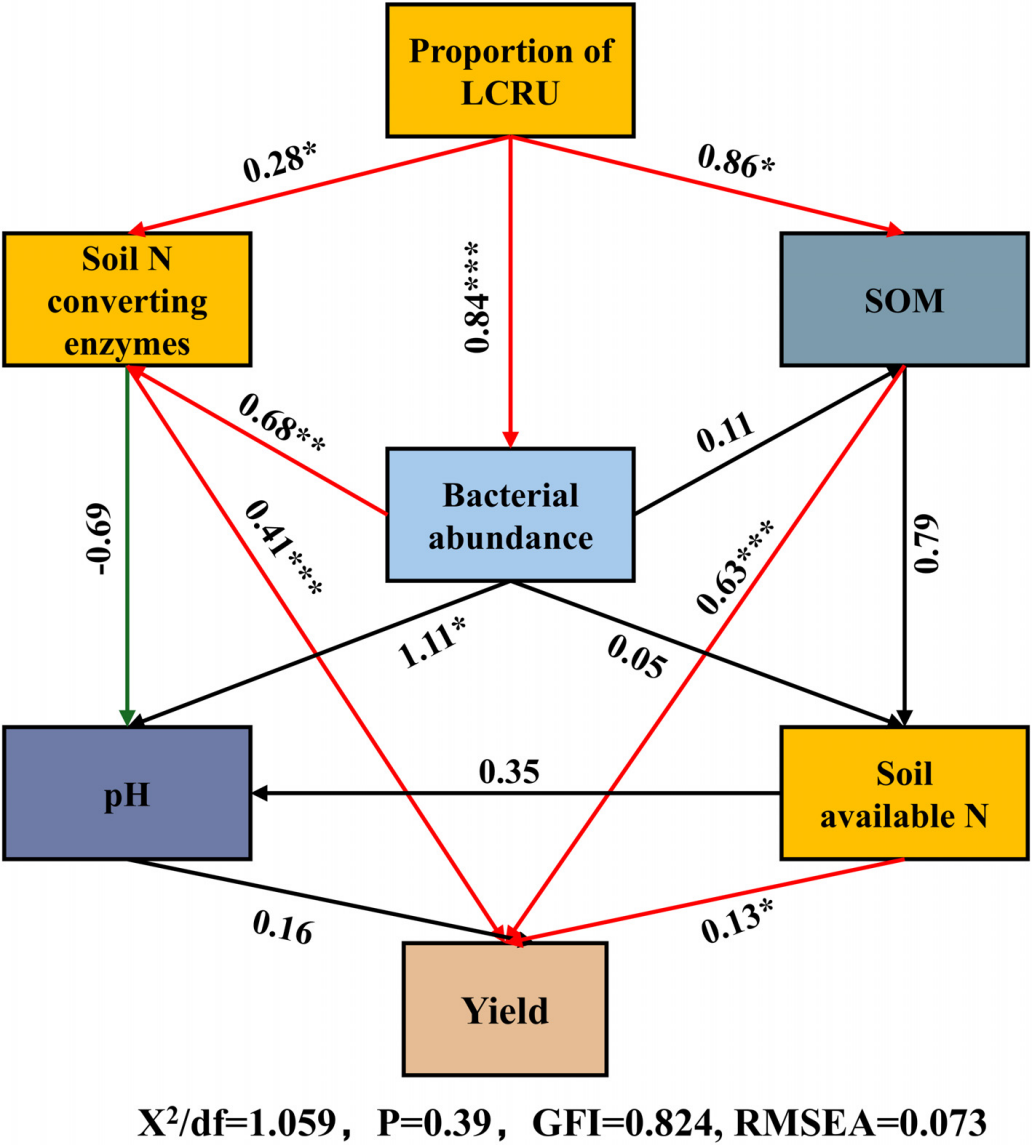
Structural equation model (SEM) analysis illustrating the effects of LCRU in fertilizer treatments on soil properties and bacterial abundance and choy sum yield. Red lines indicate positive effects, and green lines indicate negative effects. Stars denote significance at *p*<0.05, *p*<0.01, and *p*<0.001 probability levels (*, **, and ***, respectively).

Structural equation modeling (SEM) was employed to examine the impact of bacterial abundance (phylum-level), soil nitrogen-converting enzymes (NRA and UA), soil organic matter (SOM), pH, and available nitrogen in the soil (including NO_3_^−^-N and NH_4_^+^-N) on the yield of choy sum, seeking to investigate their potential interrelationships (**Figure 8**). The results demonstrated a substantial positive correlation between LCRU and soil nitrogen-converting enzymes (*p*<0.05). Bacterial abundance exhibited a notable positive association with choy sum yield through its influence on soil nitrogen-converting enzymes (*p*<0.01) and likewise positively affected available nitrogen in the soil, which had a significant positive effect on choy sum yield in turn (*p*<0.05). Moreover, soil available nitrogen was significantly correlated with choy sum yield (*p*<0.05). Furthermore, nutrients released from the decomposition of SOM supported beneficial microbial communities, thereby improving the soil’s ability to retain moisture and nutrients. Soil available nitrogen was positively influenced by the bacterial abundance and yet negatively affected by soil pH, and it had a direct positive effect on choy sum yield. Overall, the application of LCRU stimulated bacterial growth by enhancing soil nutrients and activating enzymes involved in carbon and nitrogen cycling, ultimately leading to an increased choy sum yield.

## 4 Discussion

### 4.1 Application of LCRU to promote the growth of choy sum and improve its yield and quality

In CRFs, nutrient particles are encapsulated by carrier molecules through specialized coating materials, which regulate nutrient release into agricultural soil. These coating agents, frequently referred to as excipients, are particularly designed to synchronize nutrient availability with the crop’s specific needs (Liu et al., 2019; Liu et al., 2022). In comparison to conventional fertilizers, CRFs have lower water solubility and a slower nutrient release rate in the soil, thereby reducing nutrient loss and improving the efficiency of a single application. In this study, a sustainable provision of soil nutrients by LCRU was observed, supporting the established principles of nutrient-release mechanisms in association with CRFs. Field experiments showed significant enhancements in plant height, stem diameter, and SPAD values of choy sum treated with LCRU. These findings are consistent with previous studies that have examined the impact of controlled-release fertilizers on plant growth metrics (Salimi et al., 2023; Li L. et al., 2019; Tian et al., 2016). Furthermore, an increase in the dry weight of aboveground choy sum was detected on the condition that LCRU was applied, echoing the results of Wu et al. (2019), where polymer-coated urea was illustrated to enhance rice yields. Quality improvements in crops were also observed; previous research has emphasized increases in vitamin C and soluble sugar content resulting from the utilization of CRFs. (Supplement Govil et al. 2024; Dong et al., 2016; Qu et al., 2020). Similar outcomes were observed in this study, where LCRU elevated the levels of vitamin C, soluble sugar, and soluble protein in choy sum. Additionally, a comparative analysis with conventional urea revealed that LCRU substantially optimized the absorption of N, resulting in a marked increase in nitrogen fertilizer use efficiency. This finding aligns with the results of Chu et al. (2005), who reported comparable improvements in nitrogen uptake and efficiency through the deep application of controlled-release urea in maize crops. In conclusion, the evidence from this study, corroborated by existing literature, suggests that LCRU not only promotes plant growth and nutrient uptake but also improves crop quality.

### 4.2 Applying LCRU improved soil nutrient, enzyme activities, and bacterial diversity increased choy sum yield

LCRU gradually releases nitrogen in the soil, continuously providing the nutrients needed for crop growth. This slow-release characteristic enhances nitrogen utilization efficiency and has a comprehensive regulatory effect on the physical and chemical properties, enzyme activity, microbial communities, and crop yield of the soil Simultaneously, controlled-release nitrogen fertilizer, by gradually releasing nitrogen, minimizes abrupt changes in soil pH, thereby maintaining the acid-base balance of the soil. This study has determined that the nutrient release pattern of LCRU in the soil aligns with the growth cycle of choy sum, affecting each other in several aspects and thus promoting the growth of choy sum. The gradual release feature of LCRU positively impacts the soil’s physical and chemical properties by providing a continuous supply of available nitrogen (AN), thereby enhancing soil fertility. This is the main reason why LCRU and LCRU15 treatments indicated higher levels of available nitrogen, NO_3_^−^-N, and NH_4_^+^-N in the soil compared to CU, as confirmed by previous research (Shaji et al., 2021). Additionally, Chen et al. (2022) likewise corroborated these findings in a related study on soil NO_3_^−^-N and NH_4_^+^-N levels following the application of coated urea. Nonetheless, the rapid dissolution of CU in soil water results in the volatilization of N_2_O and NH_3_, nitrate leaching, and a reduction in available nitrogen. The sustained release of LCRU, which increases the urea content in the soil, is the main reason for the observed rise in UA and NRA, as noted in previous studies (Zhang et al., 2017). Moreover, the slow-release characteristics of LCRU provide an optimal nutrient concentration for a longer period, stabilizing nitrogen supply, which activates enzymes related to nitrogen metabolism, including UA and invertase, promoting the decomposition and mineralization of organic matter, thereby enhancing soil fertility (Tian et al., 2019). Additionally, the gradual release of controlled-release nitrogen fertilizer reduces nitrogen loss during nitrification and denitrification processes, enhancing nitrogen utilization efficiency. Consequently, LCRU and LCRU15 improved soil carbon and nitrogen cycling, with the most significant effects on bacterial community structure, leading to an increase in soil bacterial diversity (Gao et al., 2022). This result is mainly attributed to the slow-release nature of LCRU, which offers a consistent substrate for nitrogen-related enzymes in the soil, maintaining and enhancing enzyme activity, thereby increasing nutrient availability and avoiding excessive or insufficient nitrogen impacts on microbial communities. Except for that, the lignin-based coating material utilized in this study acts as a carbon source that can support microorganisms during its degradation. In contrast, the rapid release of traditional nitrogen fertilizers may result in short-term spikes in soil nitrogen concentration, inhibiting certain sensitive microbial communities (Beltran-Garcia et al., 2021). As Zeng et al. (2016) suggested, lower nitrogen input rates can have a positive influence on soil bacterial community composition. In this study, correlation analysis revealed a significant positive relationship between bacterial phyla (Actinobacteriota, Bacteroidota, Gemmatimonadota, Firmicutes, Cyanobacteria, Nitrospirota, and Bdellovibrionota) and soil indicators including NRA, UA, RA, AN, NO_3_^−^-N, and NH_4_^+^-N. This correlation is mainly attributed to the slow and sustained nutrient release of LCRU, which balanced the nutrient elements in the soil, leading to changes in enzyme activity and bacterial diversity. Meanwhile, this finding aligns with research conducted by Shen et al., 2022 which reported that controlled-release fertilizers exhibit a slow nutrient-release profile. Additionally, it was observed that an increase in nitrogen content corresponded with a reduction in bacterial abundance, a finding consistent with the work of Wang et al. (2023), which indicated that microbial responses to nitrogen addition are frequently inconsistent. In summary, the application of LCRU significantly enhances soil fertility and bacterial abundance, with a 15% reduction in the LCRU formulation (LCRU15) producing the most beneficial effects.

In conclusion, LCRU influences crop growth and yield by regulating soil physical and chemical properties, enzyme activity, and microbial communities. Besides, the sustained low-level nitrogen supply from LCRU optimizes the root growth environment, enhances the availability of other nutrients in the soil (including phosphorus, potassium, and trace elements), and promotes comprehensive nutrient absorption by crops. Moreover, a positive correlation was observed between soil available nitrogen, enzyme activity, microbial activity, and crop yield. The improvement in enzyme activity and microbial diversity indicates a more dynamic nutrient conversion process in the soil, ensuring an adequate nutrient supply for crops and contributing to increased yield.

## 5 Conclusion

In this study, it was noted that the application of LCRU and LCRU15 caused significant increases in plant height, stem diameter, SPAD values, and above-ground dry weight of choy sum. Quality parameters of choy sum, including vitamin C, soluble sugar, and soluble protein content, were also determined to be enhanced by LCRU and LCRU15 treatments. Due to the controlled-release characteristics of these fertilizers, an enhanced absorption of nutrients (nitrogen, phosphorus, and potassium), especially nitrogen. In comparison to the CU, nitrogen use efficiency in choy sum increased by 64.29%-68.90% with LCRU and LCRU15 treatments, with the highest efficiency observed in the LCRU15 treatment. Soil properties were also affected by the application of LCRU and LCRU15, with increases in soil-available nitrogen, NO_3_^−^-N content, NH_4_^+^-N content, urease, and nitrate reductase activities observed than CU-treated soil. Nonetheless, no significant differences were observed in soil pH and organic matter content between LCRU, LCRU15, and CU treatments. Moreover, soil fertility and bacterial diversity were positively impacted by LCRU and LCRU15, with a 15% reduction in LCRU demonstrating the most significant effect. Higher levels of various bacterial phyla, such as Actinobacteriota, Bacteroidota, Gemmatimonadota, Firmicutes, Cyanobacteria, Nitrospirota, and Bdellovibrionota, were observed, along with an increase in the abundance of genera like Sphingomonas, Flavisolibacter, Saccharimonadales, Streptomyces, and Bryobacter. Notably, bacterial abundance was higher in the LCRU15 treatment than in LCRU. In conclusion, this study demonstrates that lignin-based controlled-release urea can be adopted to regulate soil nutrients and soil bacterial diversity to increase choy sum yields. These findings establish a theoretical foundation for the use of biomass-based controlled-release fertilizers in sustainable agricultural practices.

## Data Availability

The original contributions presented in the study are included in the article/**Supplementary Material**. Further inquiries can be directed to the corresponding author.

## References

[B1] Abalos D. Recous S. Butterbach-Bahl K. Notaris C. D. Rittl T. F. Topp C. F. E.. (2022). A review and meta-analysis of mitigation measures for nitrous oxide emissions from crop residues. Sci. Total Environ. 828, 154388. doi: 10.1016/j.scitotenv.2022.154388 35276154

[B2] Bajwa D. S. Pourhashem G. Ullah A. H. Bajwa S. G. (2019). A concise review of current lignin production, applications, products and their environmental impact. Ind. Crop Prod. 139, 111526. doi: 10.1016/j.indcrop.2019.111526

[B3] Beltran-Garcia M. J. Martínez-Rodríguez A. Olmos-Arriaga I. Valdes-Salas B. Mascio P. D. White J. F. (2021). Nitrogen fertilization and stress factors drive shifts in microbial diversity in soils and plants. Symbiosis. 84, 379–390. doi: 10.1007/s13199-021-00787-z

[B4] Channab B. E. El Idrissi A. Zahouily M. Essamlali Y. Whitet J. C. (2023). Starch-based controlled release fertilizers: a review. Int. J. Biol. Macromol. 238, 124075. doi: 10.1016/j.ijbiomac.2023.124075 36940767

[B5] Chen J. Lu S. Zhang Z. Zhao X. Li X. M. Ning P.. (2018). Environmentally friendly fertilizers: a review of materials used and their effects on the environment. Sci. Total Environ. 613-614, 829–839. doi: 10.1016/j.scitotenv.2017.09.186 28942316

[B6] Chen X. J. Guo T. Mo X. Zhang L. D. Wang R. F. Xue Y. N.. (2023). Reduced nutrient release and greenhouse gas emissions of lignin-based coated urea by synergy of carbon black and polysiloxane. Int. J. Biol. Macromol. 231, 123334. doi: 10.1016/j.ijbiomac.2023.123334 36682667

[B7] Chen X. J. Guo T. Yang H. C. Zhang L. D. Xue Y. N. Wang R. F.. (2022). Environmentally friendly preparation of lignin/paraffin/epoxy resin composite-coated urea and evaluation for nitrogen efficiency in lettuce. Int. J. Biol. Macromol. 221, 1130–1141. doi: 10.1016/j.ijbiomac.2022.09.112 36113589

[B8] Chu H. Y. Hosen Y. Yagi K. Okada K. Ito O. (2005). Soil microbial biomass and activities in a Japanese andisol as affected by controlled release and application depth of urea. Biol. Fert Soils. 42, 89–96. doi: 10.1007/s00374-005-0011-3

[B9] Dong Y. J. He M. R. Wang Z. L. Chen W. F. Hou J. Qiu X. K.. (2016). Effects of new coated release fertilizer on the growth of maize. J. Plant Nutr. Soil Sc. 16, 637–649. doi: 10.4067/S0718-95162016005000046

[B10] Fertahi S. Ilsouk M. Zeroual Y. Oukarroum A. Barakat A. (2021). Recent trends in organic coating based on biopolymers and biomass for controlled and slow-release fertilizers. J. Control Release. 330, 341–361. doi: 10.1016/j.jconrel.2020.12.026 33352245

[B11] Frank L. A. Onzi G. R. Morawski A. S. Pohlmann A. R. Guterres S. S. Contri R. V. (2020). Chitosan as a coating material for nanoparticles intended for biomedical applications. React Funct. Polym 147, 104459. doi: 10.1016/j.reactfunctpolym.2019.104459

[B12] Gao Y. X. Song X. Zheng W. K. Wu L. Chen Q. Yu X. J.. (2022). The controlled-release nitrogen fertilizer driving the symbiosis of microbial communities to improve wheat productivity and soil fertility. Field Crops Res. 289, 108712–108712. doi: 10.1016/j.fcr.2022.108712

[B13] Govil S. Long N. V. D. Escribà-Gelonch M. Hessel V. (2024). Controlled-release fertiliser: Recent developments and perspectives. Ind. Crop Prod. 219, 119160. doi: 10.1016/j.indcrop.2024.119160

[B14] Huber T. Mussig J. Curnow O. Panget S. S. Bickerton S. Staiger M. P. (2012). A critical review of all-cellulose composites. J. Mater Sci. 47, 1171–1186. doi: 10.1007/s10853-011-5774-3

[B15] Iqbal A. Ali I. Yuan P. Khan R. Liang H. Wei S. Q. (2022). Combined application of manure and chemical fertilizers alters soil environmental variables and improves soil fungal community composition and rice grain yield. Front. Microbiol. 13. doi: 10.3389/fmicb.2022.856355 PMC9330912 35910624

[B16] Li H. M. Qi Y. G. Zhao Y. X. Chi J. M. Cheng S. J. (2019). Starch and its derivatives for paper coatings: a review. Prog. Org Coat. 135, 213–227. doi: 10.1016/j.porgcoat.2019.05.015

[B17] Li L. Tian B. Li L. Shi M. Guan Y. Liu H. (2019). Preparation and characterization of silicone oil modified polyurethane damping materials. J. Appl. Polym Sci. 136, 47579. doi: 10.1002/app.47579

[B18] Li Z. Qiu L. Zhang T. E G. Y. Zhang L. L. Wang L. L.. (2023). Long-term application of controlled-release potassium chloride increases maize yield by affecting soil bacterial ecology, enzymatic activity and nutrient supply. Field Crop Res. 297, 108946. doi: 10.1016/j.fcr.2023.108946

[B19] Liu J. L. Yang Y. C. Gao B. Li Y. C. Xie J. Z. (2019). Bio-based elastic polyurethane for controlled-release urea fertilizer: fabrication, properties, swelling and nitrogen release characteristics. J. Clean Prod. 209, 528–537. doi: 10.1016/j.jclepro.2018.10.263

[B20] Liu W. Y. Price S. Bennett G. Maxwell T. M. R. Zhao C. Y. Walker G.. (2022). A landscape review of controlled release urea products: patent objective, formulation and technology. J. Control Release. 348, 612–630. doi: 10.1016/j.jconrel.2022.06.009 35709877

[B21] Melino V. J. Tester M. A. Okamoto M. (2022). Strategies for engineering improved nitrogen use efficiency in crop plants via redistribution and recycling of organic nitrogen. Curr. Opin. Biotech. 73, 263–269. doi: 10.1016/j.copbio.2021.09.003 34560475

[B22] Miyatake M. Ohyama T. Yokoyama T. Sugihara S. Motobayashi T. Kamiyad T.. (2019). Effects of deep placement of controlled-release nitrogen fertilizer on soybean growth and yield under sulfur deficiency. Soil Sci. Plant Nutr. 65, 259–266. doi: 10.1080/00380768.2019.1615827

[B23] Paraskar P. M. Prabhudesai M. S. Hatkar V. M. Kulkarni R. D. (2021). Vfron: a review. Prog. Org Coat 156, 106267. doi: 10.1016/j.porgcoat.2021.106267

[B24] Perez J. J. Francois N. J. (2016). Chitosan-starch beads prepared by ionotropic gelation as potential matrices for controlled release of fertilizers. Carbohyd Polym. 148, 134–142. doi: 10.1016/j.carbpol.2016.04.054 27185124

[B25] Qu Z. M. Qi X. C. Liu Y. L. Liu K. X. Li C. L. (2020). Interactive effect of irrigation and polymer-coated potassium chloride on tomato production in a greenhouse. Agr Water Manage 235, 117060. doi: 10.1016/j.agwat.2020.106149

[B26] Riseh R. S. Vatankhah M. Hassanisaadi M. Kennedy J. F. (2023). Increasing the efficiency of agricultural fertilizers using cellulose nanofibrils: a review. Carbohyd Polym. 321, 121313. doi: 10.1016/j.carbpol.2023.121313 37739539

[B27] Salimi M. Channab B. El Idrissi A. Zahouily M. Motamedi E. (2023). A comprehensive review on starch: Structure, modification, and applications in slow/controlled-release fertilizers in agriculture. Carbohyd Polym. 322, 121326. doi: 10.1016/j.carbpol.2023.121326 37839830

[B28] Shaji H. Chandran V. Mathew L. (2021). Organic fertilizers as a route to controlled release of nutrients//controlled release fertilizers for sustainable agriculture. Acad. Press, 231–245. doi: 10.1016/B978-0-12-819555-0.00013-3

[B29] Shen M. C. Shi Y. Z. Bo G. D. Liu X. M. (2022). Fungal inhibition of agricultural soil pathogen stimulated by nitrogen-reducing fertilization. Front. Bioeng Biotech. 10. doi: 10.3389/fbioe.2022.866419 PMC9039341 35497365

[B30] Shyamala B. N. Jamuna P. (2010). Nutritional content and antioxidant properties of pulp waste from daucus carota and beta vulgaris. Malaysian J. Nutr. 16, 397–408. doi: 10.0000/PMID22691993 22691993

[B31] Song S. W. Liao G. X. Liu H. C. Sun G. W. Chen R. Y. (2012). Effect of ammonium and nitrate ratio on nutritional quality of Chinese kale. Advanced materials Res. 461, 13–16. doi: 10.4028/www.scientific.net/AMR.461.13

[B32] Tian J. Dungait J. A. J. Lu X. Yang Y. Hartley I. P. Zhang W.. (2019). Long-term nitrogen addition modifies microbial composition and functions for slow carbon cycling and increased sequestration in tropical forest soil. Global Change Biol. 25, 3267–3281. doi: 10.1111/gcb.14750 31273887

[B33] Tian H. Y. Li Z. L. Lu P. F. Wang Y. Jia C. Wang H.. (2021). Starch and castor oil mutually modified, cross-linked polyurethane for improving the controlled release of urea. Carbohyd Polym. 251. doi: 10.1016/j.carbpol.2020.117060 33142612

[B34] Tian S. Q. Xue X. A. Wang X. W. Chen Z. C. (2022). Preparation of starch-based functional food nano-microcapsule delivery system and its controlled release characteristics. Front. Nutr. 9. doi: 10.3389/fnut.2022.982370 PMC9421261 36046140

[B35] Tian C. Zhou X. Liu Q. Peng J. W. Wang W. M. Zhang Z. H.. (2016). Effects of a controlled-release fertilizer on yield, nutrient uptake, and fertilizer usage efficiency in early ripening rapeseed (brassica napus l.). J. Zhejiang Univ-Sc B. 17, 775–786. doi: 10.1631/jzus.B1500216 PMC5064171 27704747

[B36] Vejan P. Khadiran T. Abdullah R. Ahmad N. (2021). Controlled release fertilizer: a review on developments, applications and potential in agriculture. J. Control Release. 339, 321–334. doi: 10.1016/j.jconrel.2021.10.003 34626724

[B37] Verma K. K. Song X. P. Degu H. D. Guo D. J. Joshi A. Huang H. R.. (2023). Recent advances in nitrogen and nano-nitrogen fertilizers for sustainable crop production: a mini-review. Chem. Biol. Technol. Ag. 10, 111. doi: 10.1186/s40538-023-00488-3

[B38] Wang X. D. Feng J. G. Qin Q. K. Han M. G. Shen Y. W. Liu M. L.. (2023). Globally nitrogen addition alters soil microbial community structure, but has minor effects on soil microbial diversity and richness. Soil Biol. Biochem. 179, 108982. doi: 10.1016/j.soilbio.2023.108982

[B39] Wu Q. Wang Y. H. Ding Y. F. Tao W. K. Gao S. Li Q. X.. (2019). Effects of different types of slow- and controlled-release fertilizers on rice yield. J. Integr. Agr. 20, 1503–1514. doi: 10.1016/S2095-3119(20)63406-2

[B40] Yang S. H. Peng S. Z. Xu J. Z. He Y. P. Wang Y. J. (2015). Effects of water saving irrigation and controlled release nitrogen fertilizer managements on nitrogen losses from paddy fields. Paddy Water Environ. 13, 71–80. doi: 10.1007/s10333-013-0408-9

[B41] Zeng J. Liu X. Song L. Lin X. Zhang H. Shen X.. (2016). Nitrogen fertilization directly affects soil bacterial diversity and indirectly affects bacterial community composition. Soil Biol. Biochem. 92, 41–49. doi: 10.1016/j.soilbio.2015.09.018

[B42] Zhang Y. Hao X. Alexander T. W. Thomas B. W. Shi X. J. Lupwayi N. Z. (2018). Long-term and legacy effects of manure application on soil microbial community composition. Biol. Fert Soils. 54, 269–283. doi: 10.1007/s00374-017-1257-2

[B43] Zhang J. J. He P. Ding W. C. Ullah S. Abbas T. Li M. Y.. (2021). Identifying the critical nitrogen fertilizer rate for optimum yield and minimum nitrate leaching in a typical field radish cropping system in China. Environ. pollut. 268, 115004. doi: 10.1016/j.envpol.2020.115004 33010674

[B44] Zhang J. S. Wang C. Jing B. L. Liang T. Y. He J. Xiang H.. (2017). Effects of controlled release blend bulk urea on soil nitrogen and soil enzyme activity in wheat and rice fields. J. Appl. Ecology. 28, 1899–1908. doi: 10.13287/j.1001-9332.201706.003 29745152

[B45] Zhong K. Lin Z. T. Zheng X. L. Jiang G. B. Fang Y. S. Mao X. Y.. (2013). Starch derivative-based superabsorbent with integration of water-retaining and controlled-release fertilizers. Carbohyd Polym. 92, 1367–1376. doi: 10.1016/j.carbpol.2012.10.030 23399166

