# Supplementary material for: Lignin-based controlled-release urea improves choy sum growth by regulating soil nitrogen nutrients and bacterial diversity
Source: Front Plant Sci. 2024 Dec 4;15:1488332. doi: 10.3389/fpls.2024.1488332 (PMC11652836; doi:10.3389/fpls.2024.1488332)
Supplement: Supplementary file 1 [file DataSheet1.docx]

***Supplementary Material***

**Lignin-based controlled-release urea improves Chinese flowering cabbage growth by regulating soil nitrogen nutrients and** **rhizosphere bacterial diversity**

**Xiaojuan Chen^1^& Bosi Lu^2^, Bowen lv^1^, Shaolong Sun^2*^**

*1.* College of Agriculture, Guangxi University, Nanning, Guangxi, Guangxi Key Laboratory for Agro-Environment and Agro-Products Safety, China

*2.College of Natural Resources and Environment, South China Agricultural University, Guangzhou, Guangdong, China*

*** Correspondence author:**

Shaolong Sun

[sunshaolong328@scau.edu.cn](mailto:sunshaolong328@scau.edu.cn).

8 pages including 1 Figure.

11 pages 1 Figure and 1 Table


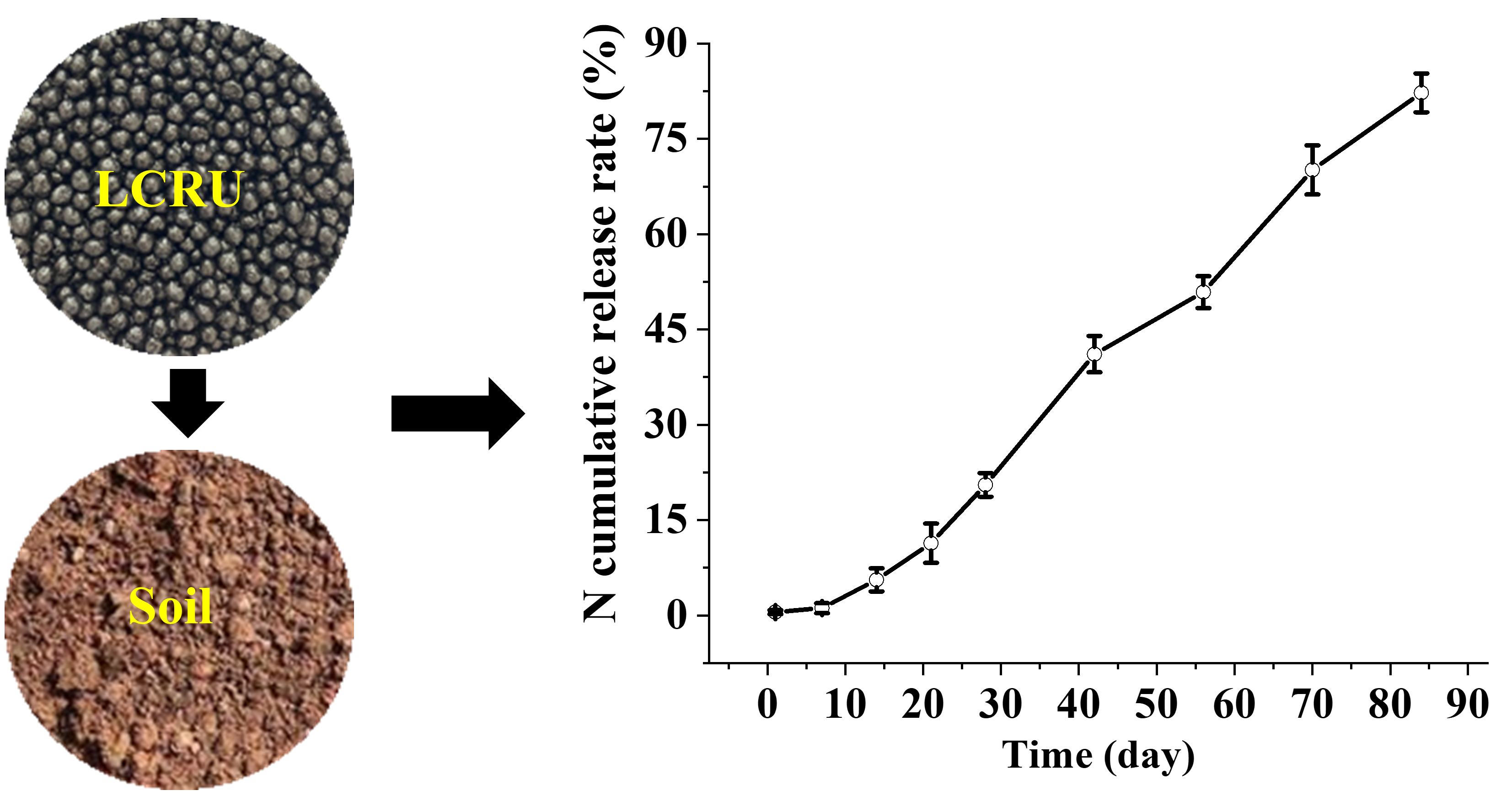


Figure1 Slow-release curve of LCRU in soil

Table1 Amplification primer sequence

| Amplified region | Primer sequence |
| --- | --- |
| 16S rDNA V3-V4 (338-806) | ACTCCTACGGGAGGCAGCAG |
|  | GGACTACHVGGGTWTCTAAT |


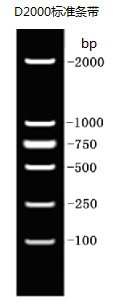


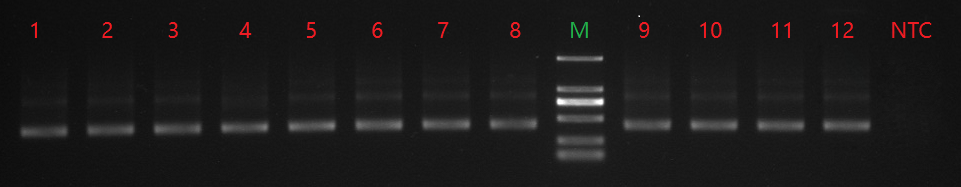


Figure 2 Agarose gel electrophoresis test results
